# When I say … entrustability

**DOI:** 10.1111/medu.14005

**Published:** 2019-11-19

**Authors:** Olle ten Cate

**Affiliations:** ^1^ Center for Research and Development of Education University Medical Center Utrecht Utrecht The Netherlands

## Abstract

In this latest contribution to the "When I say…" series, the author clarifies defining features of the words 'entrustable' and 'entrustability' while highlighting confusions that can arise when used differently.

In 2005, I introduced the word 'entrustability' in a brief paper about competency‐based postgraduate training in this journal. The title was '*Entrustability of Professional Activities and Competency‐Based Training*'.^1^ Although entrustability was a neologism, its meaning was not difficult to understand. Entrustable was coined as a property of a professional activity, qualifying the activity suitable for entrustment decisions for trainees.

Now, almost 15 years later, entrustable professional activities (EPAs) are becoming mainstream terminology in competency‐based education for the health professions. Although this is a very exciting observation, I have also observed how the words entrustable and entrustability have started to be used with different meanings. Language is a living thing and nobody ‘owns’ a word nor has the power to control the habits of its use. But the linguistic shift of entrustable to characterise things other than activities may be questioned. Entrustability was not meant to qualify learners, for example, as ‘entrustable’ or ‘pre‐entrustable learners’,^2^, ^3^ and in expressions such as a ‘pathway to entrustability’ (https://medicine.yale.edu/tlc/MedEdDay/pastMedEd/2015/Moadel%2520poster_225082_284_23458_v1.pdf), or to qualify scales, as in ‘entrustability scales’.^4^

Let me explain why I avoid using the word ‘entrustability’ for learners or scales.

With EPAs and entrustment as emerging concepts in the assessment of medical trainees,^5^ there is a wish to characterise learners who can be trusted to execute a critical health care activity versus those who cannot (yet). ‘Trustworthy’ versus ‘untrustworthy’ is clearly not very attractive terminology, as these words have a too general, psychological and emotional connotation. For that reason, authors have creatively started qualifying learners as ‘pre‐entrustable’ versus ‘entrustable’. Subsequently, others have taken this up to start creating scales for entrustability of learners. Why could that be problematic?

To be entrusted with an activity or responsibility concords with the Oxford Dictionary's meaning of entrustment to '*assign the responsibility for doing something to someone*' or '*to put something into someone's care or protection*'.^6^ It is an act of choice by the trustor: one can make an ‘entrustment decision’ or choose not to. If learner entrustability were a continuous scale, any point on that scale could be available to qualify a learner. That principle does not concur with the idea of entrustment decision making. Responsibility is given or not given; supervision is direct (in the room; proactive) or indirect (not in the room; at some distance). The analogy of the driver's license makes clear that there is no ‘1.7’ or ‘2.4’ score on a 5‐point scale for ‘entrustability’ to drive. Either the pupil is deemed ready to drive with an instructor or is deemed ready to drive without an instructor. The instructor could potentially sit on the back seat and be available to give instructions but not to take over. Sitting there would be an in‐between scale position, but would still require a discrete decision. ‘Entrustment and supervision scales’, or just ‘entrustment scales’ (which are terms I prefer to ‘entrustability’ scales), are ordinal, non‐continuous scales, as they focus on decisions and link to discreet levels of supervision. A more extensive explanation is provided elsewhere.^7^

The confusion may stem from the distinction between competencies and EPAs, which I often discover is not clear to everyone. Competencies are person descriptors, as they signify what individuals are able to do, whereas EPAs are work descriptors and only reflect the work, tasks and activities that are to be carried out in health care irrespective of who does that work. Competence of an individual (in general or for something specific) may be depicted on a scale, with anchors derived from a Dreyfus progression (novice, advanced, competent, proficient, expert)^8^ or any other model. Entrustment does not translate to a continuous scale. If ‘entrustability’ were to be a scale that is not directly linked to decisions of entrustment, it may become another proficiency scale. Such scales already exist; I do not think we need a new one.

So, I avoid the word ‘entrustability’ to qualify a learner. Although ‘trustworthy’ describes a person and sounds like an alternative option, it is not an elegant one in EPA‐based assessment language. I therefore regularly use ‘readiness’ as a better, less confusing alternative. ‘Not yet ready’ for a new task or responsibility sounds much better than ‘not yet trustworthy’. Likewise, ‘entrusting’ a learner with an activity is to be preferred to ‘trusting’ a learner with an activity. Entrustment is naturally linked with an object, such as an EPA or a patient; trust is a more general verb that may have an object but does not require one.

When I say ‘entrustability’, it has a restricted meaning. I will observe this evolution of language as others continue to employ different meanings. Language holds power to organise our world through social interaction, but it may also confuse when what I say is not what you hear.^9^ We should be as precise as possible. Language is an instrument with inherent limitations, but we have no alternative way to share our thinking, and we must use it to clarify thoughts as best we can. Medical Education's *When I Say* series offers an excellent opportunity to share such clarifications.

There is, however, a bigger reason for all of this. That is to contribute to a conversation about trust in the worlds of health care and education: worlds which seem to be moving in a direction of assessment and control, a direction that reflects distrust rather than trust. Talking about trust, entrustment and entrustability can hopefully redirect this trend.
